# A Bayesian Belief Network model to link sanitary inspection data to drinking water quality in a medium resource setting in rural Indonesia

**DOI:** 10.1038/s41598-020-75827-7

**Published:** 2020-11-02

**Authors:** D. Daniel, Widya Prihesti Iswarani, Saket Pande, Luuk Rietveld

**Affiliations:** Department of Water Management, Delft University of Technology, Delft, The Netherlands

**Keywords:** Environmental sciences, Risk factors, Civil engineering

## Abstract

Assessing water quality and identifying the potential source of contamination, by Sanitary inspections (SI), are essential to improve household drinking water quality. However, no study link the water quality at a point of use (POU), household level or point of collection (POC), and associated SI data in a medium resource setting using a Bayesian Belief Network (BBN) model. We collected water samples and applied an adapted SI at 328 POU and 265 related POC from a rural area in East Sumba, Indonesia. Fecal contamination was detected in 24.4 and 17.7% of 1 ml POC and POU samples, respectively. The BBN model showed that the effect of holistic—combined interventions to improve the water quality were larger compared to individual intervention. The water quality at the POU was strongly related to the water quality at the POC and the effect of household water treatment to improve the water quality was more prominent in the context of better sanitation and hygiene conditions. In addition, it was concluded that the inclusion of extra “external” variable (fullness level of water at storage), besides the standard SI variables, could improve the model’s performance in predicting the water quality at POU. Finally, the BBN approach proved to be able to illustrate the interdependencies between variables and to simulate the effect of the individual and combination of variables on the water quality.

## Introduction

Water quality has a prominent place in the Sustainable Development Goal 6.1^1^, because it has been recognised that unsafe drinking water is responsible for high numbers of diarrheal morbidity and mortality among children below the age of five^1^. Water quality analysis becomes important because supplied water, especially in low and middle-income countries (LMICs), is often contaminated, even though it is categorised as an improved water source^2^. Groundwater, which is considered safer than surface waters, is also found contaminated in many locations^3^. In Addition, high levels of contamination has been found at the household level in LMICs and water quality often deteriorates after collection^4–6^.

To tackle this, the World Health Organization (WHO) and International Water Association (IWA) launched a Water Safety Plan (WSP) concept, which is a comprehensive risk assessment and management covering all steps in water supply from catchment to consumers^7^. The goal is to minimise the risk of contamination and provide safe drinking water to people. Identifying potential sources of contamination is part of the risk assessment and one of the critical elements in WSP.

In order to assess potential sources of contamination in a water supply system, systematic observation, called sanitary inspections (SI), are performed. SI variables record potential sources of contamination based on “on-site inspection and evaluation by qualified individuals of all conditions, devices, and practices in the water-supply system that pose an actual or potential danger to the health and well-being of the consumer”^8^. SI have the advantage to be easy to implement, not expensive, can be adapted to the local context, and can give a quick snapshot of potential causes or pathways of contamination. However, SI are not a substitute for drinking water quality testing, but identify contamination source in the system, especially in the context of risk management, and can be used to design appropriate actions to change the situation^9^. Therefore, it has been recommended to accompany drinking water quality testing with SI^10^.

Conducting drinking water quality testing in LMICs, however, can be challenging, especially because of limited resources such as laboratory facilities or infrastructure^11^. Bain et al.^12^ summarised all available microbial water quality tests for low and medium resource settings and they classified the resource settings into low, medium, and high resource settings. A low resource setting has been characterised as having no laboratory equipment and 24 h electricity; the medium one having at least a basic laboratory or clean space with 24 h electricity; while the high resource setting is equipped with reliable 24 h electricity and a modern laboratory. Researchers are able to choose relevant water quality tests according to local context or situation.

Attempts have been made to link SI data to drinking water quality in order to be able to judge the reliability of the system. The most common approach has been to analyse the SI and drinking water quality by using statistical analyses, e.g., bivariate correlation or regression analyses, especially in high resource settings^6,10,13–16^.

Bayesian Belief Network (BBN) is another alternative to analyse factors responsible for the water quality^17,18^. BBN offers benefits compared to other statistical methods, such as the ability to integrate quantitative and qualitative information in the model and an intuitive visualisation of the hypothetical causal relationships that can aid stakeholders with less technical knowledge in understanding the system^19^.

However, the application of BBN in analysing water quality at the household level [mentioned as a point of use (POU)] and at water source or point of collection (POC) is very limited. Hall and Le^20^ utilised BBN to predict the faecal contamination of drinking water by household’s socio-economic characteristics as predictor variables, however not using SI variables. To the authors’ knowledge , the present study is the first to link drinking water contamination at the POU with a *combination* of water quality at POC, the hygiene conditions in the household, water handling, and household water treatment (HWT) practices in a medium resource setting. This study aims to delineate the microbial water quality and general sanitary conditions in POC and POU in the district of East Sumba, Indonesia.

## Methods

### Study setting

**Figure 1:**
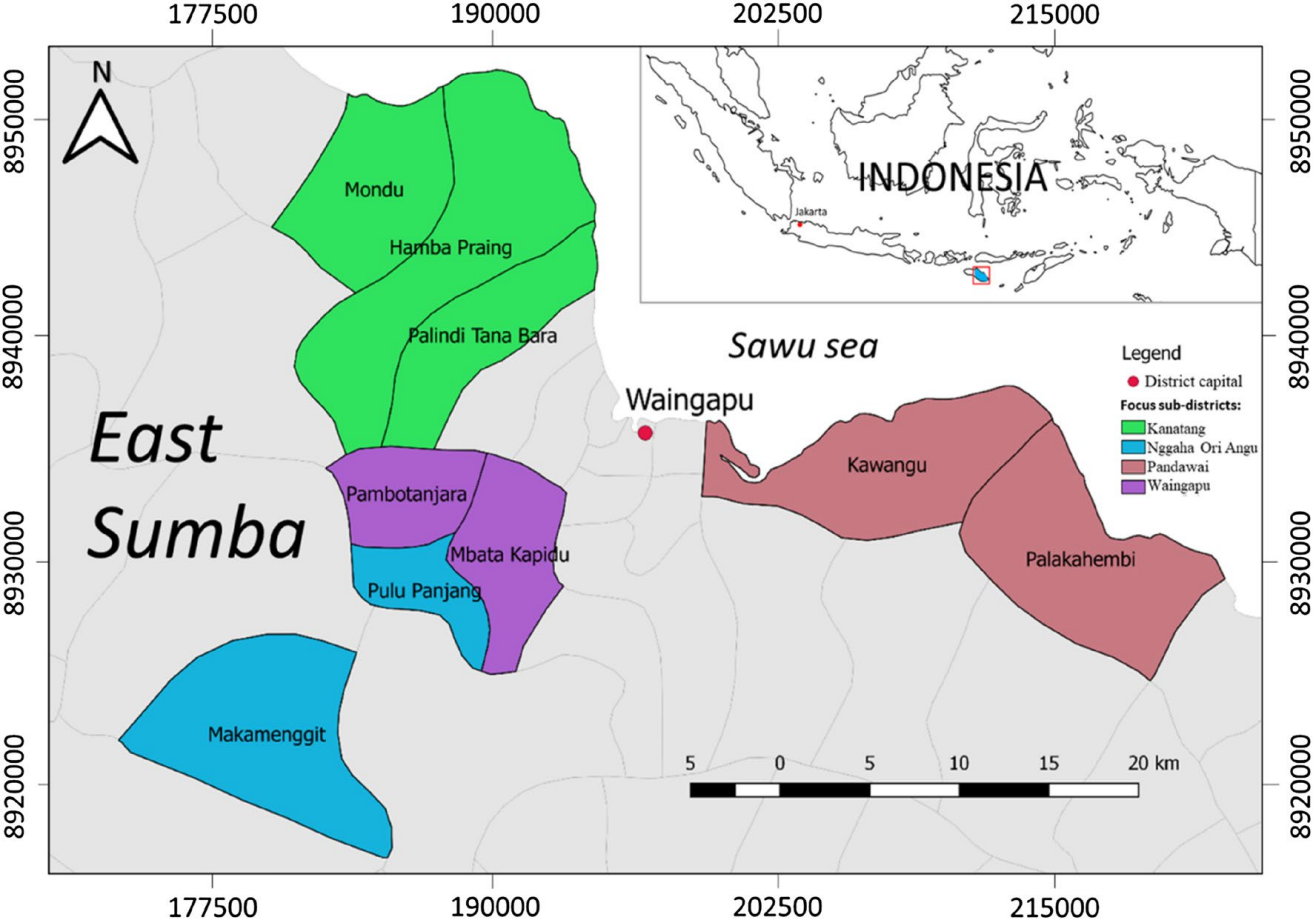
Map of the study location. There were nine villages visited in four sub-districts. The map is drawn using QGIS^24^.

A cross-sectional study was conducted in July–August 2019 in the district of East Sumba, Province East Nusa Tenggara, Indonesia (Fig. 1). This study is the continuation of a previous household water treatment study conducted in the same area^21^. A total of 328 households in nine villages in four sub-districts were revisited during this study. This area is known as one of the poorest areas in Indonesia where open defecation is still common and there is high prevalence of children’s malnutrition^22^. The topography of the area is hilly. Furthermore, about 40% the total populations in East Sumba relied on wells as their main water source and only 18% had access to piped distribution system in 2017^23^. No water treatment is conducted in the rural piped distribution systems in this area.

Approximately 100 ml of drinking water sample, i.e., from the drinking water storage container, was taken at each household. The households were asked to give water in the same way as for drinking water. The water samples were put in Nasco Whirl–Pak bags and kept inside a thermos during the transport to the field lab. All the samples were analysed within six hours after collection. We only analysed the microbial water quality and used *E. coli* as an indicator bacteria for fecal contamination in water^25^. We took 1 ml of sample using a 1 ml sterile pipette and placed it on a Nissui Compact dry EC plate (CDP) and incubated for 24 h at 35 ± 2 °C^26^. After incubation, we counted the colony forming units (CFU) of *E. coli* in the CDP and reported in concentration units (CFU/1 ml). The process was conducted as sterile as possible to prevent contamination from sample processing, e.g., using hand gloves and sterile pipette tips when processing the sample, avoid touching the inside of the whirl-pack bag when collecting and processing the sample, and working in a stable and clean space. The sample processing was conducted by two master students from Delft University of Technology who were familiar with microbial water quality analyses. According to the classification of Bain et al.^12^, our analysis was categorised as medium resource setting, e.g., there was neither distilled water and proper disinfection for laboratory equipment. Data were collected during the dry season with temperature in that area ranging from 25 to 26 °C.

For the SI, we used the Open Data Kit (ODK) software on a smartphone, and the data were transferred to a computer for analysis. We did SI at POCs and POUs. Information taken at a POC and POU can be found in Table 1. Participation was voluntary and a written informed consent was obtained from all participants. The study was approved by the Human Research Ethic Committee of Delft University of Technology and the Agency for Promotion, Investment, and One-Stop Licensing Service at the district level. All experiments were conducted in accordance with relevant guidelines and regulations.

**Table 1:** Information used for the analysis.

Point of collection (POC)^b^	Surrounding environment–hygiene condition	Water storage condition and HWT
*Type of POC* [Which source do you use for drinking water purpose right now?]^b^	*Still practise open defecation* [What types of toilet do you have?]	*Storage covered* [Is the water storage being covered (at that time)?]
*Livestock nearby* [Is there livestock near the point of collection (POC), 10 m?]	*Livestock nearby* [Is there livestock around the house?]	*Storage cracked* [Is the container cracked?]^a^
*Prone to erosion* [Is the area uphill from the source visibly eroded or prone to erosion?]	*Floor cleanliness* [How is the cleanliness of the house floor?]^a^	*Place of storage* [When not in use, is the storage container kept in a place where it may become contaminated? E.g., can be reached by animal easily; open space (risk by flies), etc.]
*Excreta / garbage nearby* [Is excreta or garbage found within 10 m of the tap stand/water source?]	*Faeces around* [Is there human or animal faeces in the yard (or even inside the house)?]^a^	*fullness level of water at storage* [How full is the water storage?]^a,c^
*Proper fencing* [Is there proper fencing or a barrier around the well to prevent contact with animals?]	*Garbage around* [Is there garbage around the house?]	Household water treatment [Is the water in the storage treated?]
*Latrine within 10 m* [Distance to the nearest latrine (m)]	*Flies around* [Could you see flies around the water storage container?]	
*Cracked structure* [Are there any damages/cracks in the system/source?]		
*E. coli detected at POC/well*^b^		

^a^The sentence inside the [ ] were the questions in the sanitary inspection and the italic words were the variable/node name in the BBN.

^b^Based on water quality testing.

^c^External variable besides standard SI variables.

### Bayesian Belief Network (BBN)

BBN is a directed acyclic graph showing a hypothetical causal relationship between “causal” variables (where the arrow start; called “parent nodes” in BBN) and an “affected” variable (called “child node”)^27^. The strength of the relationship between parent and child node is shown by the values in the Conditional Probability Tables (CPT) of the child node. The CPT values are showing the probability of a child node will be in a particular state or category, given all possible combination of the states of its parent nodes. The CPT values can be obtained from expert or stakeholder judgment or elicitation, the output of other models or calculations, or by direct measurement. Cain^19^ provides a good and clear explanation of using a BBN in the water sector.

### Data analysis

**Figure 2:**
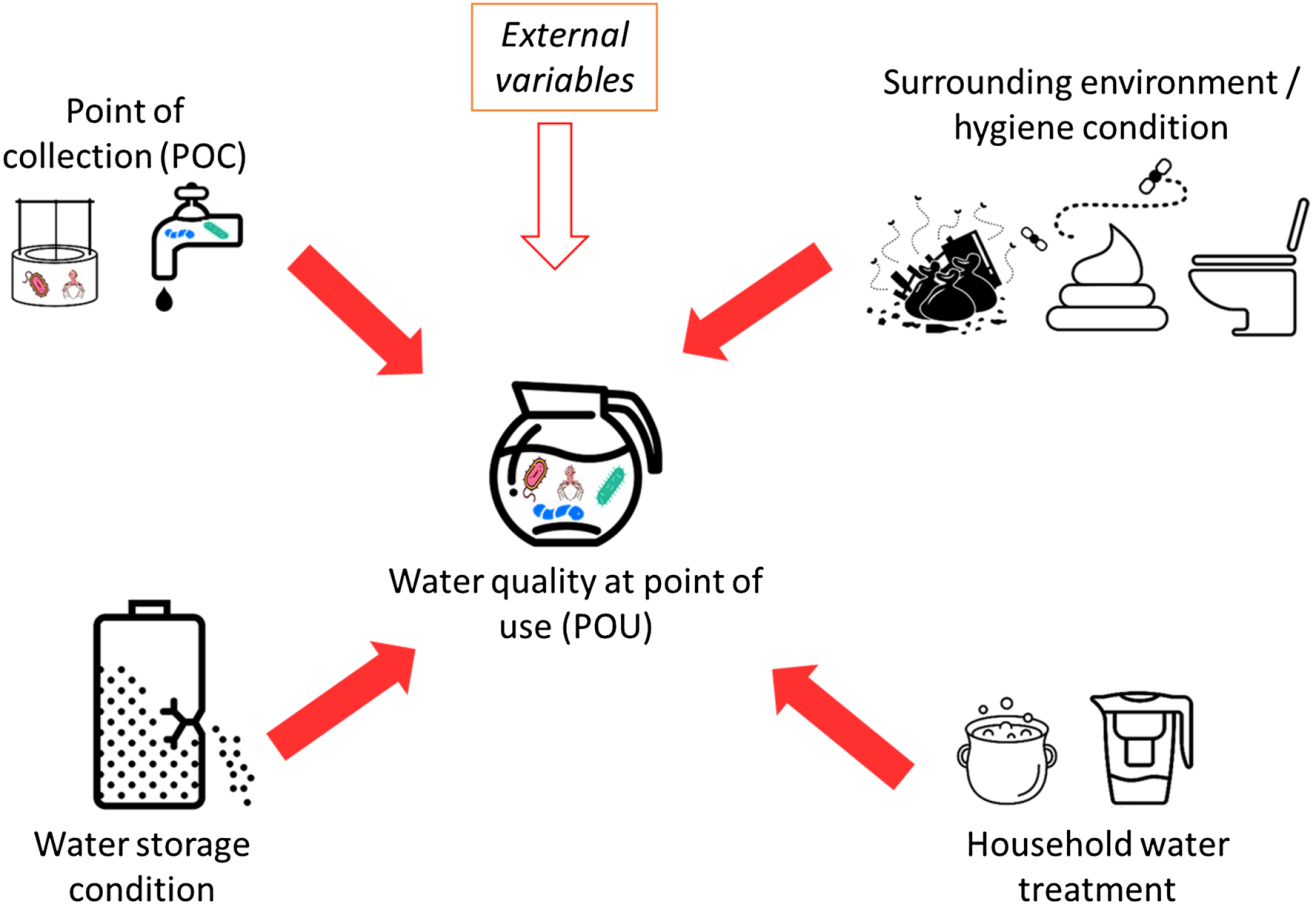
The conceptual model of five clusters of the determinants of water quality at a point of use (POU). Red arrows indicate that the variables are often included in a standard SI form and white arrow is not included in the standard SI form.

A BBN’s structure is often inspired by a conceptual theory or framework or by consensus between experts in that field^28^. There are some conceptual frameworks from previous water, sanitation, and hygiene (WASH) studies that can be adapted into a BBN’s structure^29,30^, including the well-known F-diagram ^31^. According to those frameworks, there are four main clusters of determinants of water quality at POU: (1) Surrounding environment–hygiene condition, (2) HWT, (3) (the water quality at) POC, and (4) the water storage conditions (see Fig. 2). All variables for these four cluster are often included in a standard SI form^8^.

However, Navab-Daneshmand et al.^29^ argues that fecal contamination at the household level in LMICs is complex. This implies that there might be other variables, besides SI variables, that could correlate with the household drinking water quality, such as container material, duration of storing water, inappropriate extraction water from storage, etc^32–34^. However, all these “external” factors are not included in the standard SI form^8^.

Based on the above mentioned literature, we created a conceptual model of potential factors that could influence the water quality at the household level (Fig. 2). The conceptual model includes multiple contamination pathways in a system^35^ and was used to create the BBN’s structure by clustering SI variables based on those five clusters.

**Figure 3:**
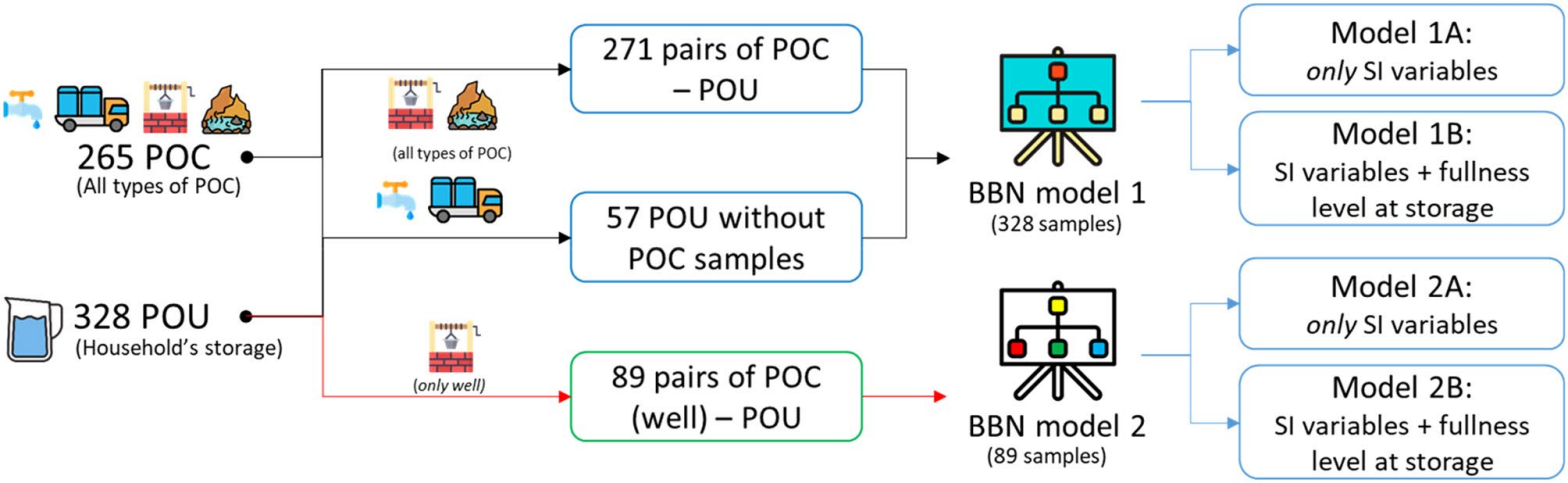
Overview of the datasets and analysis.

Because some houses used the same POC, we could make pairs of 271 POCs–POUs (Fig. 3). 49 POU did not have POC samples, i.e., POC samples were not taken, mostly due to long distance walk (> 30 min return trip). However, these 49 POU samples were included in the BBN analysis, since the EM algorithm compensated for the missing information with the available data^36^.

**Figure 4:**
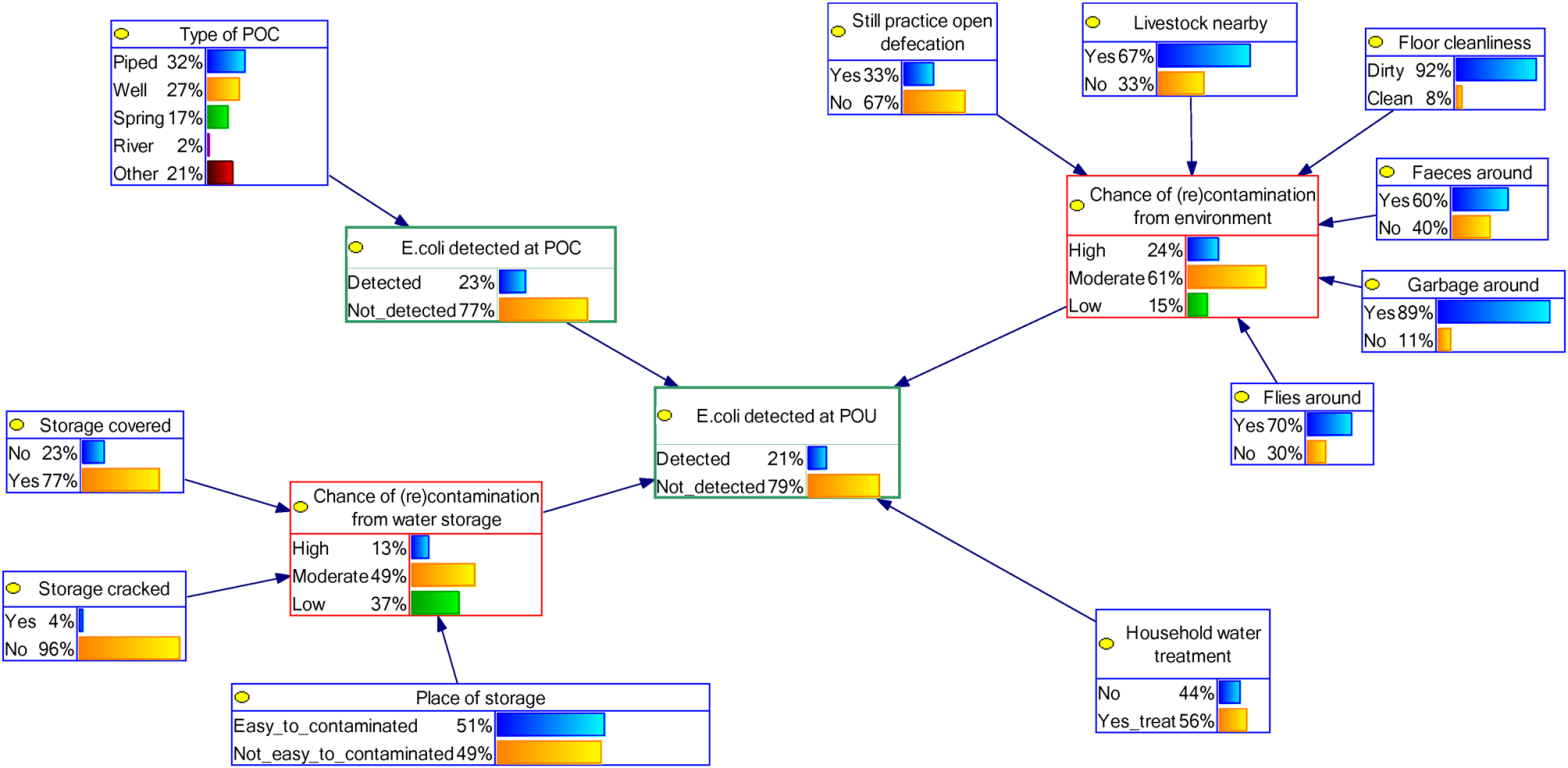
BBN model 1A (type of POC as a parent node of “*E. coli* detected at POC”). Blue nodes: data obtained from SI; green nodes: data obtained from water quality testing; red nodes: intermediate nodes were obtained by summation of the value in the outer nodes. The percentages in each node indicate the probability of a node being in a certain state, e.g., 56% of the households perform household water treatment.

**Figure 5:**
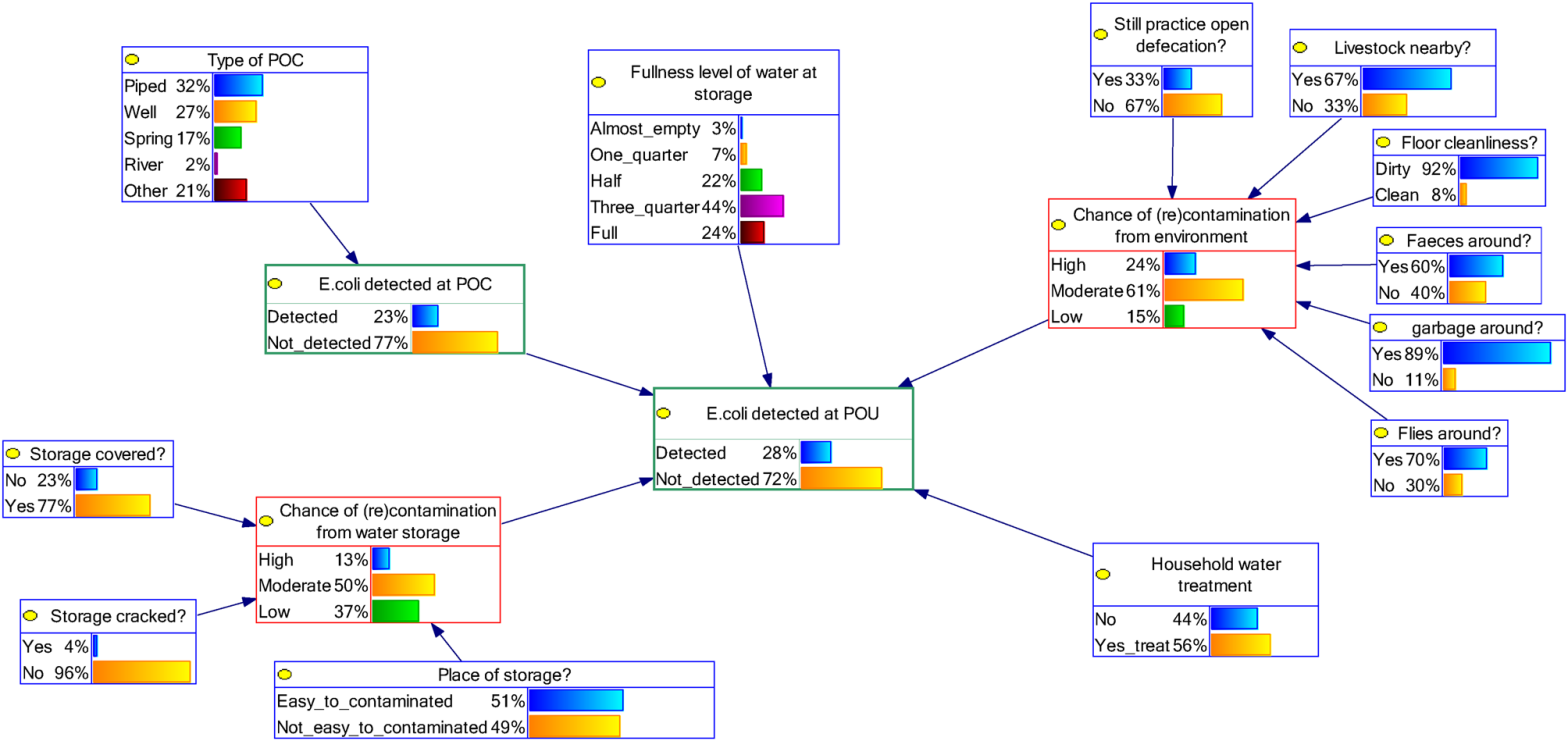
BBN model 1B (type of POC as a parent node of “*E. coli* detected at POC” and adding node “fullness of water at storage” as one of the parent nodes of “*E. coli* detected at POC”).

**Figure 6:**
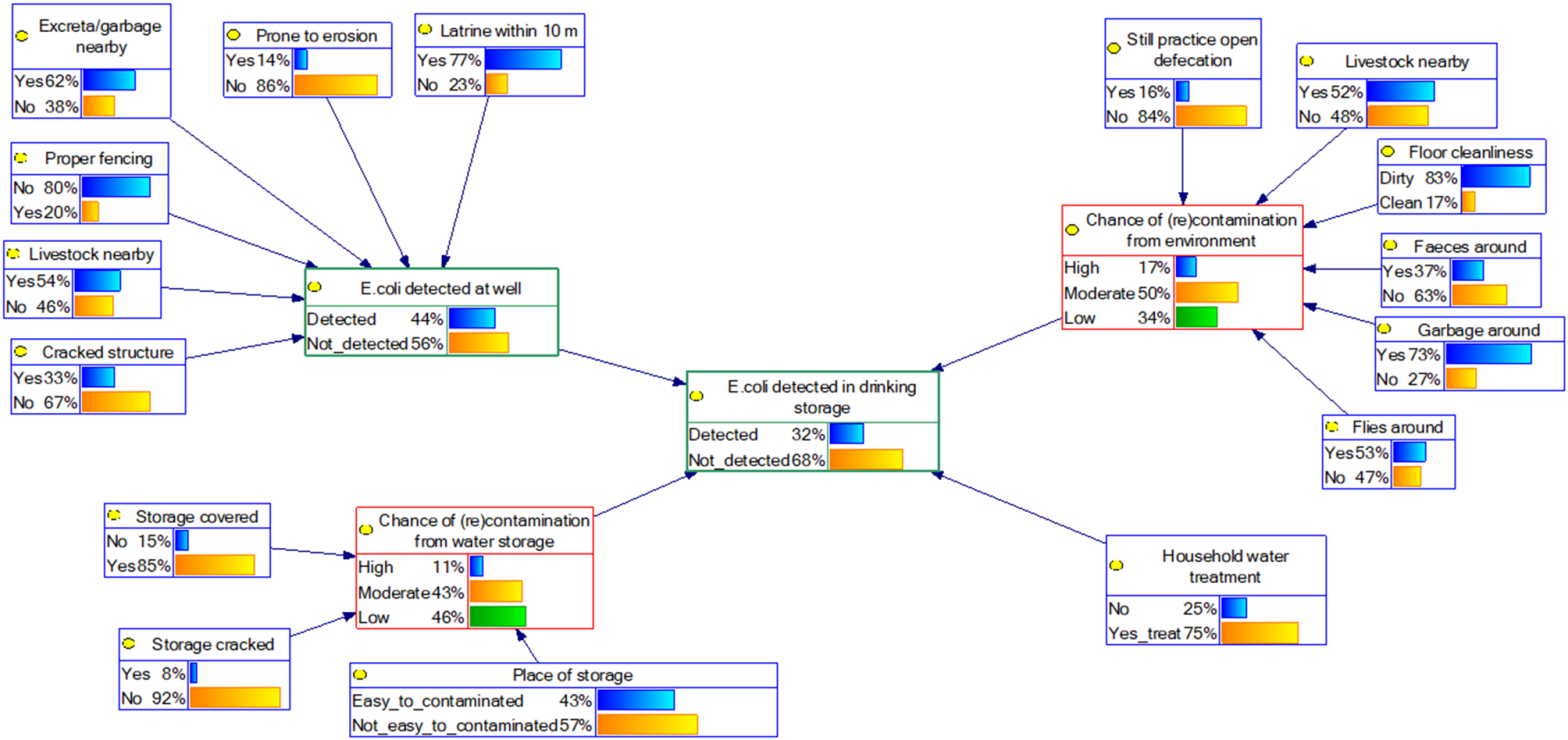
BBN model 2A (SI variables at well as parent nodes of “*E. coli* detected at POC”).

**Figure 7:**
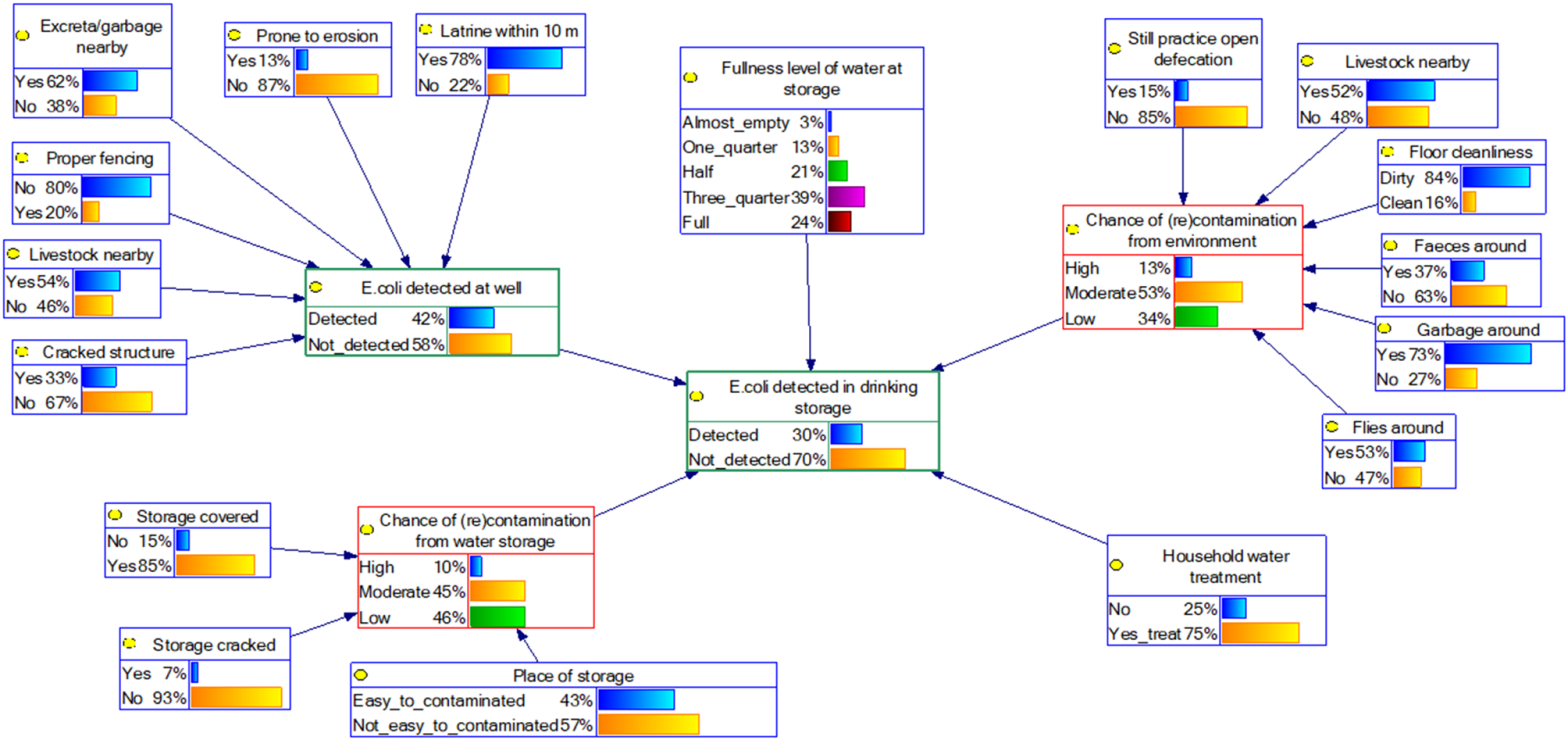
BBN model 2B (SI variables at well as parent nodes of “*E. coli* detected at POC” and adding node “fullness of water at storage” as one of the parent nodes of “*E. coli* detected at POC”).

Four BBN models of the water quality at the POU were created (Fig. 3). BBN model 1 (A and B) and 2 (A and B) differ in terms of the variables used in the cluster of POC. For BBN model 1 we added node *Type of POC* as a parent node for *E. coli detected at POC* (Figs. 4, 5). But for BBN model 2 we used information of the SI at the POC as parent nodes of *E. coli detected at POC*, but we modelled only one type of POC: well (Figs. 6, 7). That is because the SI information that we collected at POC were only relevant to the well’s characteristics. For BNN model 1, we had in total of 328 samples and for BNN model 2 was only 89 well samples (Fig. 3).

In addition, we added one extra variable, *fullness level of water at storage*, on top of both models and compared the model’s performance, i.e., BBN model 1A vs 1B and model 2A vs 2B. This variable could indicate the duration of storing water, because water quality could deteriorate over time^4^. Thus, BBN model 1A and 2A were the BBN models with SI variables *only* and BBN model 1B and 2B were the BBN models with SI variables plus variable *fullness level of water at storage*. The results of validation tests, i.e., AUC value, indicated the model’s performance. The predictive inference tests were then conducted using BBN models with the best performance.

Moreover, Since it is not recommended to have many parent nodes in BBN^19^, we needed to reduce the BBN structure as much as possible. Clustering the SI variables reduces the parent nodes of the outcome node, e.g. water quality at the POC. All variables in the SI for POC were grouped as one cluster and the variables in the SI related to water storage were grouped as another cluster. In the latter case, e.g., three variables related to the condition of the water storage, *Storage covered*, *Storage cracked*, and *Place of storage*, were connected to an intermediate node *Chance of (re)contamination from water storage* (red node in Fig. 4).

Since we did not have the information on intermediate nodes in our datasets, the CPT corresponding to this node was populated manually. First, we gave score 1 to the best situation in each variable, e.g., score 1 if “yes” in variable *storage covered* and score 1 if “no” in variable *storage cracked*. Then we created a simple index by summing all the scores of the three parent nodes. Finally, we categorised it as “low” if the total score was 0–1, “moderate” if the total score was 2, and “high” if the total score was 3. In the same way, another intermediate node *Chance of (re)contamination from environment* was created by six variables (six parent nodes of this variable, see Fig. 4). We categorised it as “low” if the total score was 0–2, “moderate” if the total score was 3–4, and “high” if the total score was 5–6. Different from the other intermediate nodes, we used the results of water quality testing to fill the information of node *E. coli detected at POC* (see Fig. 4; green nodes). BBN requires discrete or categorical information for the analysis. Therefore, we discretised and categorised the number of *E. coli* into *E. coli* detected or non-detected.

We used software GeNIe 2.2 (https://www.bayesfusion.com) to perform the BBN analysis. The software uses the Expectation Maximization (EM) algorithm to estimate the CPT values^36^. We performed validation tests using the same software to assess the model’s performance. We used the ten-fold cross-validation and the performance was reflected by the value of area under the ROC curve (AUC): AUC of 0.5 indicates poor model, AUC between 0.5 and 0.7 is a “less accurate” model, 0.7 < AUC ≤ 0.9 is a “moderately accurate”, 0.9 < AUC < 1 is a “highly accurate” model, and AUC = 1 is a perfect model^37^.

We also conducted a “predictive inference” in BBN, to find influential nodes that help us to prioritise actions to improve the water quality of POU in that area. We performed that by setting the state of a specific node to 100% and observe the updated probability in the output node. For example, if we wanted to observe the influence of HWT on POU’s water quality, we set the probability of node *Household water treatment* being “yes_treat” to 100% and observed the updated probability of *E. coli detected at POU* being “detected”. We did that to all states in all nodes.

Finally, we simulated the “best scenario”, i.e., targeting all SI variables or potential source of contaminations in the system, by setting the best situation of all SI variables (outer nodes) at all clusters, including node *Household water treatment* being “yes_treat” and node *E. coli detected at POC* being “not_detected”. By setting node *E. coli detected at POC* being “not_detected”, we assumed that all types of water source that household use are safe.

## Results

### Socio-demographic characteristics of the respondents

When asked about the education of the household’s head, 12.5% of them had no formal education, and 57.3%, 11.9%, and 18.3% finished primary, secondary, and higher school, respectively. In terms of housing condition, 87.6% did not have permanent walls, e.g., wood or bamboo, 7.5% did not have a permanent roof, i.e., straw, and 71.4% still had a natural floor, i.e., compacted soil. Moreover, 45.3% of the respondents had no electricity. About 32.7% of the respondents practised open defecation. Based on observations, households either had simple pit latrines or pour-flush latrines, some were communal and some were in respective households. Tap water (from a small-scale distribution network) was used by 31.8% of the respondents, followed by wells 27.2%, water trucks 19.6%, and spring water 17.4%, respectively. Remaining respondents used river water, rainwater, or refill potable water stations. Boiling was used to treat the drinking water.

### Description of the sanitary inspection and water quality results

The general hygiene situation of the respondents is depicted in the BBN model, i.e. the outer nodes in Fig. 4 (in blue colour). For example, 23% of the respondents did not cover their drinking storage and only 30% of the respondent’s houses were free from flies. From the cluster of *surrounding environment–hygiene condition*, we found that 66.7% of the respondents kept their livestock near the house, resulting in 60% of the respondents had animal faeces around the house. In addition, 89% and 70% of the respondents had garbage and flies around the water storage or house, respectively. These conditions led to only 15% respondents had low chance of contamination from the surrounding environment and hygiene condition.

The general condition of the cluster *water storage condition* indicated that 37% of the respondents had a low chance of contamination from “bad condition of water storage”, i.e., comply to all three criteria: storage with cover, without cracking, and proper-safe place. About 77% and 96% of the storages were found to be covered and without cracking, but 51% of the storages were put in a place that can be prone to (re)contamination, e.g. on the floor.

Of all the POU samples, 56.5% of the respondents claimed to treat water at the time of visit. 75% of households who abstracted water from river treated their drinking water, followed by 68.5% and 59.4% from households who used well and piped system, respectively.

Of all the POU samples, 56.3% of our respondents claimed to treat water at the time of the visit. For the water quality, we did not detect *E. coli* in the 1 ml samples in 195 (75.6%) of the POC samples and 270 (82.3%) of the POU samples. *E. coli* was not detected in almost 90% of the piped and spring samples, while 42% and 83% of well and river samples, respectively, were detected with *E. coli*.

### Comparison of the BBN models’ performance

The four BBN models are shown in Figs. 4, 5, 6 and 7. We first compared the performance of BBN models with SI variables *only* and SI variables plus extra variable *fullness level of water at storage*. The validation tests of these four BBN models gave AUC value: 0.55, 0.69, 0.71, and 0.84 for model 1A (Fig. 4), 1B (Fig. 5), 2A (Fig. 6), and 2B (Fig. 7), respectively. According to the classification of Greiner et al.^37^, model 1A and 1B were classified as “less accurate” and model 2A and 2B as “moderately accurate”.

The addition of variable *fullness level of water at storage*, which is not part of “standard” SI variables, improved the model’s performance. Therefore, we decided to use BBN model 1B (Fig. 5) and 2B (Fig. 7) for further BBN analyses, because model 1 and 2 differ in structure (Fig. 3).

### Predictive inference of the BBN models

Node *E. coli detected at POC* was the most influential node (see ∆P = 21 in Table 2—left) for the model 1B (type of POC as one of the outer nodes), i.e., the better the water quality at POC, the better the water quality at the household level or POU. Node *Type of POC* and *Fullness level of water at storage* appeared as the second most influential nodes (∆P = 17 in Table 2—left). The intermediate node *Chance of (re)contamination from the water storage* was the third most influential node (∆P = 10 in Table 2—left).

**Table 2:** Predictive inference, measuring the effect of changes in the states of each node on the output node of BNN models: *E. coli detected at POU* (drinking water storage).

BBN model 1B: with type of POC as one of the outer nodes										BBN mode 2B: with SI at well as one of the outer nodes						
Variable	Probability of *E. coli* not-detected at POU (%)								∆P^a^	Variable	Probability of *E. coli* not-detected at POU (%)					∆P
**Point of collection**										**Point of collection**						
Type of POC	Piped		Well	Spring	River		Other		17	Cracked structure	Yes		No			2
	75		69	75	58		72				69		71			
*E. coli* detected at POC	Yes			No					21	Livestock nearby	Yes		No			1
	56			77							70		71			
**Household water treatment**										Proper fencing	Yes		No			0
Household Water treatment	No			Yes					6		70		70			
	69			75						Excreta/garbage nearby	Yes		No			0
**(re)contamination from environment–hygiene condition**											70		70			
Still practise open defecation	Yes			No					2	Prone to erosion	Yes		No			1
	71			73							71		70			
Livestock nearby	Yes			No					1	Latrine within 10 m	Yes		No			0
	72			73							70		70			
Floor cleanliness	Dirty			Clean					1	*E. coli* detected at POC	Yes		No			19
	72			71							59		78			
Faeces around	Yes			No					1	**Household water treatment**						
	72			73						Household water treatment	No		Yes			13
Garbage around	Yes			No					0		60		73			
	72			72						**(re)contamination from environment–hygiene condition**						
Flies around	Yes			No					0	Still practise open defecation	Yes		No			4
	72			72							67		71			
Chance of contamination from the environment	High			Moderate		Low			7	Livestock nearby	Yes		No			5
	68			75		70					68		73			
**(re)contamination from water storage**										Floor cleanliness	Yes		No			2
Storage covered	Yes			No					5		70		72			
	74			69						Faeces around	Yes		No			5
Storage cracked	Yes			No					4		67		72			
	69			73						Garbage around	Yes		No			1
Place of storage	Easy to contaminated			Not easy to contaminated					3		70		71			
	71			74						Flies around	Yes		No			1
Chance of contamination from water storage	High			Moderate		Low			10		70		71			
	64			74		74				Chance of contamination from the environment	High	Moderate	Low			22
**Fullness level of water at storage**											57	67	79			
Fullness level of water at storage	Almost empty	One quarter		Half		Three quarter		Full	17	**(re)contamination from water storage**						
	58	64		70		75		74		Storage covered	Yes		No			0
											70		70			
										Storage cracked	Yes		No			0
											70		70			
										Place of storage	Easy to contaminated		Not easy to contaminated			2
											71		69			
										Chance of contamination from water storage	High	Moderate	Low			4
											68	72	68			
										**Fullness level of water at storage**						
										Fullness level of water at storage	Almost empty	One quarter	Half	Three quarter	Full	4
											70	73	69	70	71	

The value under each category corresponding to a node as displayed in the first column is the updated probability of the output node being “Not_detected” given that all households maintain this state. The left side of the table was for the BBN model 1A (Fig. 5) and the right side was for BBN model 2B (Fig. 7).

^a^∆P is the difference between the lowest and highest value of the updated probability of output node: *E. coli detected at POU* being “Not_detected”, in %. Examples of how to read the table: (a) row 4–5 *BBN model 1B*: if the type of POC is piped, the Probability of *E. coli* not-detected at POU (%) is 75%; (b) row 6–7 *BBN model 1B*: if *E. coli* is detected at POC (“yes”), the Probability of *E. coli* not-detected at POU (%) is 56%; (c) row 4–5 *BBN model 2B*: if there is a cracked in the structure (“yes”), the Probability of *E. coli* not-detected at POU (%) is 69%.

The probability of not detected *E. coli* at POU was 75% for households who used both *Piped* and *Spring*, considering other information in the BBN model. The fuller the level of water in the storage, the better the water quality at POU was: the probability of *E. coli* contamination at POU was 58% for *Almost empty* compared to 74% for *Full*. Among all three outer nodes in the cluster *(re)contamination from water storage*, node *storage covered* (∆P = 5 in Table 2–left) was the most influential node.

The households who claimed to do HWT have a higher chance of not to be contaminated by *E. coli* than households who claimed not doing HWT, i.e., P_Not_detected_ = 75%, P_Not_detected_ = 69%, respectively.

In model 2B, intermediate node *Chance of (re)contamination from the environment* was the most influential node among households who used a well as their water source (∆P = 22 in Table 2—right). Node *E. coli detected at POC* was the second most influential nodes (∆P = 19 in Table 2—right), followed by node *Household water treatment* (∆P = 13 in Table 2—right). In addition, the influence of node *Fullness level of water at storage* and the intermediate node *Chance of (re)contamination from the water storage* was not large, compared to model 1B (both had ∆P = 4 in Table 2—right).

The effect of HWT to improve the water quality was larger in model 2B (∆P = 13 in Table 2—right), compared to model 1B (all types of POC; ∆P = 6 in Table 2—left). If we compare the situation of intermediate nodes *Chance of (re)contamination from the environment* and *Chance of (re)contamination from the environment* in model 1B (Fig. 5) and 2B (Fig. 7), the hygiene situation was better in model 2B. The probability of being “high” in both intermediate nodes in model 2B was lower than in model 1B, e.g., 24% in model 1B compared to 13% in model 2B for the intermediate node *Chance of (re)contamination from the environment*.

Furthermore, keeping the house free from livestock (P_Not_detected_ = 73%) and faeces (P_Not_detected_ = 72%) seemed important to reduce the probability of fecal contamination at the household storage among households who used a well as their water source. Respondents who practiced open defecation had a larger probability of fecal contamination at the POU than they who did not, i.e., P_Not_detected_ = 67%, P_Not_detected_ = 71%, respectively (∆P = 4). The influence of HWT to reduce the chance of contamination was prominent in model 2B, i.e., P_Not_detected_ = 73% for households who treated their drinking water and P_Not_detected_ = 69% for not treating water.

The ∆P of intermediate nodes in both model 1B and 2B were bigger than their outer (parent) nodes. For example, in model 2B, the ∆P of 6 outer nodes in the cluster of *surrounding environment–hygiene condition* had less variation (range ∆P = 1–5) compared to the intermediate node *Chance of (re)contamination from the environment* (∆P = 22), whereas the intermediate nodes were the sum of the values in outer nodes.

**Figure 8:**
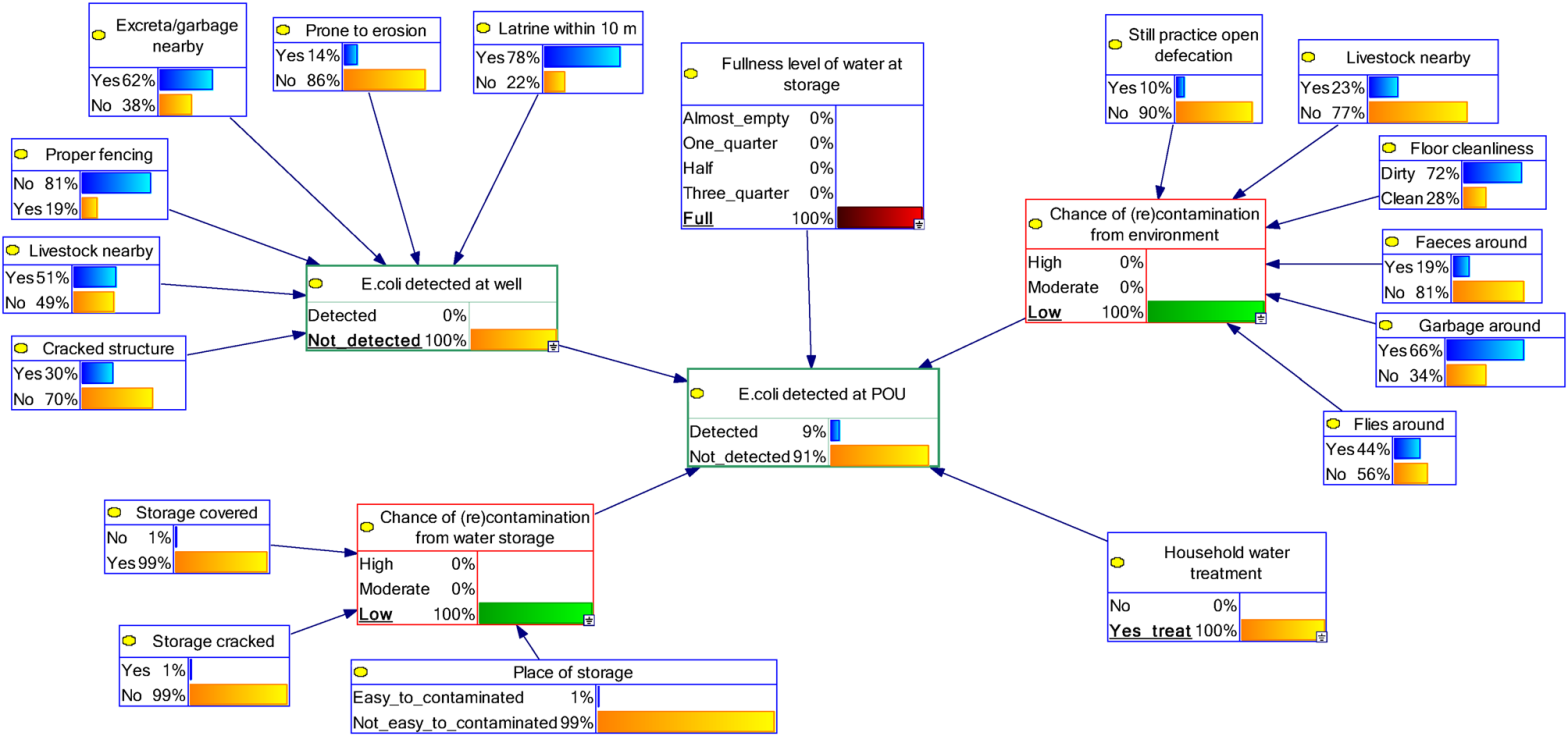
The best scenario of water and hygiene management at households level using BBN model 2B (SI at well as one of the outer nodes, SI variables, and *fullness of water at storage*).

For simulating the best scenario, i.e., combination of variables, model 2B was used to simulate all respondents (Fig. 8). The updated probability of outcome node *E. coli detected at POU* being “not_detected” was 91%, compared to the 70% in the baseline situation (Fig. 7). Given the same scenario in model 1B, the updated probability of the outcome node was 92%, compared to the 72% in the baseline (Fig. 5), which suggests the same pattern as model 2B.

## Discussion

### BBN model’s performance

Since there is no BBN study which links SI and water quality data, we compared our models’ performance with statistical analysis. Snoad et al.^13^ utilized logistic regression to predict the fecal contamination by SI and their AUC values were low (range 0.41–0.64). Other authors also used multiple statistical analyses and found that SI variables could not explain well the water quality^10,14,16^, which imply that our models (with AUC values of 0.69 and 0.84) were slightly better in predicting the water quality at POU, using SI data.

However, we found that an “external” factor, besides standard SI variables, increased the model’s performance, in our case we used the level of water *fullness inside the storage*, as also found to be relevant in other studies^32–34^, suggesting the need to extend the standard SI with external factors for better model performance. In addition, BBN models with SI variables at well (AUC for model 2A and 2B are 0.71 and 0.84, respectively) perform better than BBN models with different types of POC (AUC for model 1A and 1B are 0.55 and 0.69, respectively). Since the same type of POC, e.g., well, can have varying conditions, detailed information of the POC conditions can better explain the water quality than the information on the type of POC itself. This may explain why BBN models with SI variables as explanatory variables perform better than BBN models with types of POCs as explanatory variables.

### Sanitary inspection, water quality, and BBN predictive inferences

To the authors’ knowledge, this is the first study that links SI data with water quality in a medium resource setting. The BBN approach allowed the inclusion of all factors influencing the water quality at POU and grouping them in relevant clusters and pathways, as implied by other conceptual frameworks^29–31^. Furthermore, we were able the analyse the water quality at POU by considering not only the water management and hygiene situation at home, but also the broader scope, such as the situation at the water source. Moreover, the conventional statistical analysis methods, e.g., bivariate correlation or regression analyses, often quantify the effect of the individual variable on water quality, but not a combination of variables or pathways^6,10,16^. The BBN approach was able to simulate both the effects in one model and can then help to prioritise the interventions that improve the water quality at household level, i.e., either targeting one variable or combination of multiple variables.

The BBN approach also enabled the portrayal of interdependencies vividly among variables, while this interdependency have attracted the attention of WASH practitioners and experts over the past years^35^. For example, SI results revealed that there were some hygiene challenges related to livestock ownership. The majority of the respondents (67%) kept livestock in the surroundings of the house, which could be the reason why many flies (70%) and faeces (60%) were detected in our respondents’ houses (see Fig. 5 cluster *(re)contamination from environment–hygiene condition*). A study of Ercumen et al.^38^ found that the presence of animals is related to fecal contamination, and the presence of animal faeces is associated with diarrhea and stunting^39^. This could be the reason why this area was reported as one of the locations with the highest stunting levels in Indonesia^40^. To tackle these conditions is challenging, since in East Sumba livestock is a symbol of social status^41^.

Our BBN models (1B and 2B) showed that the water quality at POCs critically affected the water quality at the POU in the study area, which has also been found by others^6,42^. We also found that types of water source used by the households determine the drinking water quality that they have at home, similar to the findings in rural Honduras^43^. These data suggest that the fecal contamination at POU due to poor water quality at the water source, especially wells, is a serious problem in East Sumba, i.e., 40% the total populations in East Sumba used well as their main water source^23^.

Since we found that the effect of HWT to improve the water quality was larger in model 2B (POC = well only) compared to model 1B (all types of POC), we argue that the effect of HWT to improve the water quality is prominent in the case of better sanitation and hygiene conditions, i.e., the overall condition in model 2B was “more hygienic” than in model 1B. This result has also been suggested by a previous study^44^.

Model 1B showed that storage with full water had a better water quality than (almost) empty storage. The explanation could be that the water inside the empty storage was stored for a longer period than a fuller storage, resulting in larger risks for recontamination^4^ and permitting bacteria regrowth^45^.

Furthermore, we found that the ∆P (the difference between the lowest and highest value of the updated probability of output node: *E. coli detected at POU* being “Not_detected” given the specific condition of a specific node) of intermediate nodes are larger than the influence of their outer (parent) nodes. This implies that collective information of the specific cluster was more meaningful, i.e., more sensitive, to predict the water quality than individual information of specific node or variable. Additionally, it suggests that our simple index, by summing the scores of the parent nodes to populate the CPT in some intermediate nodes, was “acceptable”, i.e. simplifying the BBN structure and the intermediate nodes were related to the output node.

A previous WASH study found that a combined HWT, sanitation, handwashing, and house’s cleanliness intervention have the same effect as with HWT intervention alone in reducing fecal contamination in household drinking water^46^. In contrast to their study, we found that a combined improvement, targeting all potential contamination sources from the water source until house, had a larger effect in reducing the chance of fecal contamination in the water storage rather than the improvement of one single condition. This suggests that a holistic approach or multi-barrier prevention are needed to minimise drinking water contamination at the POU in rural households^7,47^. However, considering the costs and time constraint, based on the results on impact of water quality at POU, it can be suggested to prioritize the improvement of the water quality at the water source, based on e.g. BNN modelling. Afterwards, WASH behavioural change promotion, e.g., promoting the correct and sustained use of HWT and safe storage container, could be conducted.

Future water quality studies in that area should analyze and include other external factors that may influence the water quality at POC and POU, e.g., type and depth of the well and the types of water containers used by households. This can improve our understanding of water quality in this area.

## Conclusion

This paper introduces an application of BBN to analyse how water quality at the point of use is related to the water quality at the point of collection and associated sanitary inspection data in the medium resource settings in low-middle income countries. The model simulations showed that holistic—combined interventions improved the water quality considerably compared to individual interventions. Moreover, the results demonstrate that water quality at the POC was, as expected, related to the water quality at the POU and (correct and regular) household water treatment had a larger effect of improving the storage water quality in the case of better sanitation and hygiene conditions. We also found that the BBN model performance increased by adding an external variable besides standard SI variables, suggesting that the current SI form should accommodate more (relevant) variables. Additionally, *E. coli* was detected in 24.4 and 17.7% of POC and POU samples, respectively, and there was a hygiene issue related to the ownership and presence of livestock surround the house. Based on the water quality analysis, tap and spring water are relatively cleaner than other types of water sources and, therefore, should be prioritised by the households as main drinking water sources. In order to improve the drinking water quality in this area, reducing the contamination risk at the water source and promoting correct and regular household water treatment are suggested. From the study it can finally be concluded that the BBN approach could be considered as an alternative for conventional statistics to link sanitary inspection and water quality data in low-middle income countries.
